# Red Sea Bream Iridovirus Kinetics, Tissue Tropism, and Interspecies Horizontal Transmission in Flathead Grey Mullets (*Mugil cephalus*)

**DOI:** 10.3390/ani13081341

**Published:** 2023-04-13

**Authors:** Kyung-Ho Kim, Gyoungsik Kang, Won-Sik Woo, Min-Young Sohn, Ha-Jeong Son, Mun-Gyeong Kwon, Jae-Ok Kim, Chan-Il Park

**Affiliations:** 1 Department of Marine Biology & Aquaculture, Institute of Marine Industry, College of Marine Science, Gyeongsang National University, 2, Tongyeonghaean-ro, Tongyeong 53064, Republic of Korea; 2 Aquatic Disease Control Division, National Fishery Products Quality Management Service, 216, Gijanghaean-ro, Gijang, Busan 46083, Republic of Korea; 3 Aquatic Disease Control Division, National Fishery Products Quality Management Service, 17, Jungnim 2-ro, Tongyeong 53019, Republic of Korea

**Keywords:** horizontal transmission, virus shedding, virus kinetics, histopathological analysis

## Abstract

**Simple Summary:**

Red sea bream iridovirus (RSIV) can infect more than 30 species of farmed fish and has been reported in several Asian countries. Previous studies have reported the detection of RSIV in flathead grey mullets (*Mugil cephalus*), but research on pathogenicity, histopathological lesions, viral shedding, and the effects of infected fish on other species is very limited. In this study, we analyzed pathogenicity and viral dynamics in flathead grey mullets through immersion infection, simulating natural conditions of RSIV, and confirmed the risk of horizontal transmission to other fish species through cohabitation. Our data verify that RSIV is pathogenic to flathead grey mullets and that there is a potential for spreading the disease from fish farms to other species. Additionally, our findings revealed a high correlation between viral load and histopathological lesions in fish.

**Abstract:**

Red sea bream iridovirus (RSIV) causes significant economic losses in the aquaculture industry. We analyzed the pathogenicity of RSIV in flathead grey mullets (*Mugil cephalus*), the correlation of histopathological lesions, and interspecies horizontal transmission, through immersion infection and cohabitation challenges. Flathead grey mullets, which were challenged by immersion infection, exhibited mortality at 14 and 24 days after RSIV exposure. Viral shedding in seawater peaked 2–3 days before or after the observed mortality. Specific lesions of RSIV were observed in the spleen and kidney, and the correlation between histopathological grade and viral load was the highest in the spleen. In a cohabitation challenge, flathead grey mullets were the donors, and healthy rock bream, red sea bream, and flathead grey mullets were the recipients. Viral shedding in seawater was the highest in flathead grey mullet and rock bream at 25 °C, with 10^6.0^ RSIV copies L/g at 14 dpi. No mortality was observed in any group challenged at 15 °C, and no RSIV was detected in seawater after 30 dpi. The virus shed from RSIV-infected flathead grey mullets caused horizontal transmission through seawater. These findings suggest that rapid decision-making is warranted when managing disease in fish farms.

## 1. Introduction

Red sea bream iridovirus (RSIV) is a viral disease causing significant economic damage to the aquaculture industry [1]. RSIV was first discovered in Japanese red sea bream (*Pagrus major*) farms in 1990 and is the causative agent of red sea bream iridoviral disease (RSIVD) [2], one of the reportable diseases currently managed by the World Organization for Animal Health (WOAH) [1]. RSIV has been primarily reported in East and Southeast Asian countries [3,4,5,6,7], and in South Korea, it was first discovered in 1998 in rock bream (*Oplegnathus fasciatus*), where it caused substantial mortality every summer in water temperatures of 25 °C or higher [8,9]. Since then, RSIV has been detected in more than 30 fish species, and the range of susceptible species is expanding continuously [10]. The major infection sites of RSIV have been reported to be the spleen and kidney, and histopathological lesions are characterized by abnormally enlarged cells [10,11,12].

Horizontal transmission of RSIV has been demonstrated in previous studies and poses a serious problem for the aquaculture industry [13]. The primary infection route of RSIV is considered to be horizontal transmission through seawater, and viral shedding following RSIV infection has recently been reported [14,15,16]. Virus replication and shedding dynamics are key factors governing viral infectivity in the environment [17,18]. In particular, the waterborne transmission of RSIV infections among fish in net pen aquaculture systems sharing the same seawater has been reported in previous studies [14]. The optimal temperature range for RSIV replication has been reported to be 20–25 °C in both in vivo and in vitro analyses [19,20]. However, RSIV shedding has been reported in rock bream artificially infected with RSIV at the lower susceptibility temperature of 15 °C, and RSIV has been detected in seawater from red sea bream farms at a low water temperature of 11.7 °C [14,15]. It has been reported that rock bream surviving 100 days post-RSIV infection still carry the virus [20]. This suggests that the virus shed from infected fish could be a potential cause of the annual occurrence of RSIVD in the region. Recently, vaccines to prevent RSIV infection have been developed and commercialized; however, additional research is still required on their potential use in various fish species [21]. In summary, for effective disease control and to prevent the spread of disease, it is important to understand viral transmission between hosts and to detect pathogens at an early stage.

The flathead grey mullet (*Mugil cephalus*) is a major aquaculture species, with the third-largest aquaculture production in South Korea. Previous studies have reported the detection of RSIV in both wild and cultured flathead grey mullets [22,23,24]. Although WOAH recognizes the flathead grey mullet as an RSIV-susceptible fish species [1], no study has reported on the risk to other fish species through horizontal transmission or provided pathogenic information from RSIV infection experiments. In this study, mortality and viral shedding in seawater were confirmed through RSIV immersion infection in flathead grey mullets. In immersion-infected flathead grey mullets, histopathological scoring and infection grade criteria were defined according to RSIV infection kinetics. In addition, a cohabitation challenge model confirmed that horizontal transmission between species occurs in RSIV-infected flathead grey mullets through seawater. Our findings provide novel insights into the risk of RSIV-infected flathead grey mullets and can help in establishing measures to control RSIVD in fish farms.

## 2. Materials and Methods

### 2.1. Experimental Fish and Virus

The flathead grey mullets (total length: 10.2 ± 0.9 cm, weight: 9.2 ± 2.7 g), rock bream (total length: 10.4 ± 2.7 cm, weight: 22.7 ± 7.3 g), and red sea bream (total length: 9.7 ± 0.5 cm, weight: 13.7 ± 1.7 g) were purchased from hatcheries in Geoje and Namhae, Gyeongsangnam-do, Korea, where RSIVD was not reported; they were acclimated in a 1600 L tank for 2 weeks. Individual tanks (1600 L) of each fish were placed in a flow-through aquaculture system (500–1000 L/h) and continuously supplied with sand-filtered, 50 μm filter-housed, UV-treated (>30 mW/cm^2^) seawater during acclimation. Water temperature was maintained at 25 ± 2 °C and commercial feed was provided twice, daily. Prior to experimentation, 15 fish from each fish species were randomly selected and confirmed to be free of RSIV by polymerase chain reaction (PCR) analysis as described in the Aquatic Animal Diagnostic Test Manual for WOAH and qPCR [1,25].

In this study, the RSIV genotype II (accession number: AY532608) was utilized [15]. The virus was propagated using the *Pagrus major* fin (PMF) cell line maintained at 25 °C with L-15 medium (Gibco, Billings, MT, USA) containing 10% fetal bovine serum (Gibco), 1% antibiotic-antimycotic (100 U/mL penicillin, 100 μg/mL streptomycin, and 25 μg/mL amphotericin B, Gibco) [26]. RSIV was inoculated onto a confluent PMF cell monolayer and incubated at 25 °C for viral replication. Culture supernatants exhibiting complete cytopathic effects (CPE) were harvested and centrifuged at 10,000× *g* for 10 min at 4 °C. Subsequently, the supernatant was collected, and the viral copy numbers were determined on the basis of the method described in a previous study [25]. The virus was stored at −80 °C until further use.

### 2.2. Experimental Immersion Infection of Flathead Grey Mullets

All experimental protocols followed the guidelines set by the Institutional Animal Care and Use Committee of Gyeongsang National University (approval number: GNU-220526-E0056; GNU-220526-E0057; GNU-220526-E0058). Flathead grey mullets were reared in eight 50 L water tanks at 25 °C and 15 °C (*n* = 30 in each group) for two weeks. Fish in the immersion challenge group were exposed to 10^5^, 10^3^, and 10^1^ RSIV copies/mL (final infection concentration in the tank) for 150 min at 25 °C and 15 °C, respectively, after which the seawater was replaced with fresh seawater. The negative control group received no treatment (*n* = 30 in each group). Mortality patterns were observed daily for 40 days, and tissues (spleen, gills, kidneys, heart, stomach, eyes, liver, intestines, brain, skin, and muscle) from the dead fish in each group were collected for viral load analysis. Half (50%) of the rearing seawater for each group was replaced daily with sand filtration treated seawater, 1 μm housing filter, and UV (>30 mW/cm^2^). To determine the RSIV shedding kinetics, 500 mL of seawater (*n* = 3 in each group) was collected from fish tanks used to measure mortality after RSIV immersion at 3, 5, 7, 10, 14, 21, 30, and 40 days post-infection (dpi), virus concentration was determined, and qPCR was performed as described in Section 2.4.

To investigate the dynamics in the tissues of flathead grey mullets after the immersion challenge, another set of experiments was conducted in the manner described above. After immersion challenge, three fish from each tank were sampled at 3, 5, 7, 10, 14, 21, 30, and 40 days and whole blood, eyes, gills, skin, liver, spleen, kidneys, heart, and brain were collected. The collected organs were stored individually at −80 °C for viral load determination and fixed in 10% neutral-buffered formalin for histopathological analysis. 

### 2.3. Cohabitation Challenge

Before the RSIV cohabitation challenge, fish were reared in eight 100 L water tanks at 25 °C and 15 °C (*n* = 30 in each group) for two weeks. To confirm the horizontal transmission of the virus in RSIV-infected flathead grey mullets, fish were reared in cohabitation. Flathead grey mullets (donors, *n* = 30 in each group) were injected intraperitoneally (IP) with RSIV (10^6^ RSIV copies/fish), and naïve rock bream, red sea bream, and flathead grey mullets (recipients, *n* = 30 in each group) cohabited with donors. In the negative control group, flathead grey mullets (*n* = 30 in each group) injected with L-15 medium and naïve fish (*n* = 30 in each group) cohabited. The mortality of each group was observed for 40 days. Donor and recipient fish were distinguishable owing to being reared in two cages that allowed smooth seawater flow within the tank. Half (50%) of the rearing seawater for each group was replaced daily with sand filtration treated seawater, 1 μm housing filter, and UV (>30 mW/cm^2^). For the determination of RSIV shedding kinetics, 500 mL of seawater (*n* = 3 in each group) was collected from the fish tanks measuring mortality after RSIV cohabitation challenge at 1, 3, 5, 7, 10, 14, 21, 30, and 40 dpi, virus concentration was determined, and qPCR was performed as described in Section 2.4.

To investigate the dynamics in the tissues of flathead grey mullets after the immersion challenge, another set of experiments was conducted in the manner described above. Spleens and kidneys of fish (donors and recipients, *n* = 3 in each group) were sampled as previously described on 1, 3, 5, 7, 10, 14, 21, 30, and 40 days after cohabitant infection to determine the RSIV load in the fish. For histopathological observation, spleen and kidney tissues sampled at 3, 5, 7, 10, 14, 21, 30, and 40 days after RSIV intraperitoneal (IP) injection in fish (donors and recipients, *n* = 3 in each group) were fixed in 10% neutral formalin and analyzed as described in Section 2.6.

### 2.4. Nucleic Acid Extraction and qPCR

Genomic DNA was extracted from fish tissues (25–50 mg) and blood (100 μL) using an AccuPrep^®^ Genomic DNA Extraction Kit (Bioneer, Daejeon, Republic of Korea), following the manufacturer’s instructions. The extracted DNA was assessed for RSIV copy number using a previously described TaqMan-based qPCR method [25]. Briefly, the primer sets included Meg 1041F (5′-CCA CCA GAT GGG AGT AGA C-3′) forward primer, Meg 1139R (5′-GGT TGA TAT TGC CCA TGT CCA-3′) reverse primer, and Meg 1079P (5′-[FAM]-CCT ACT A[i-EBQ]CT TTG CGC CCA GCA TG-[phosphate]-3′) TaqMan probes [25]. All qPCR assays were performed on a Dice^®^ Real Time System III (Takara, Kusatsu, Japan), with an initial denaturation of 1 min at 95 °C, followed by 45 cycles of 5 s at 95 °C and 10 s at 60 °C. For each sample, the RSIV qPCR composition consisted of 5 μL DNA, 12.5 μL HS Prime qPCR Premix with UDG (2×) (Genetbio, Daejeon, Republic of Korea), final concentrations of 900 nM forward and reverse primers, and 250 nM TaqMan probes. The cycle threshold (C_t_) cut-off value for qPCR was set at 39.75, as described in a previous study [25]. During qPCR analysis, a negative control (diethyl pyrocarbonate treated water) well was included to confirm the absence of false positives in the PCR reaction. After measuring the weight (mg) of each tissue, the viral copy number was calculated per mg of tissue following the viral quantification analysis [25].

### 2.5. Virus Concentration Based on Iron Flocculation in Rearing Seawater

RSIV particles in rearing seawater were concentrated using an iron flocculation assay [25,27]. Initially, 500 mL of seawater was filtered through a 1.6 μm pore size glass microfiber filter (GF/A; Whatman, Maidstone, UK) to remove suspended particles. Subsequently, 4.83 g of iron (III) chloride hexahydrate (FeCl_3_∙6H_2_O) was dissolved in 100 mL of distilled water to form an iron chloride solution, and 50 μL of this solution was dispensed into the 500 mL of seawater to form Fe-virus flocculates. The seawater containing the iron chloride solution was then gently stirred at 20 °C (room temperature) for 1 h at 200 rpm using a magnetic stirrer. The Fe-RSIV flocculates were filtered under reduced pressure through a 0.8 μm pore size polycarbonate filter (Whatman) attached to a filter holder with a receiver (Nalgene, New York, NY, USA). The viruses collected on the filter were transferred to a 2 mL tube, and nucleic acids were extracted using an AccuPrep^®^ Genomic DNA Extraction Kit (Bioneer). The genomic copy numbers of the virus particles concentrated from seawater were determined using qPCR, as described in Section 2.4. To determine the viral shedding ratio (RSIV copies/L/g) of RSIV-infected fish, the average number of viral genome copies in seawater was divided by the average weight of fish surviving in the tank. Seawater for the analysis of the viral shedding ratio of all fish was collected from the mortality group tanks.

### 2.6. Histopathological Analysis

Histopathological analyses were conducted on all dissected organs (brain, eye, gills, heart, kidney, liver, muscle, and spleen) of the fish samples. Each sample was fixed in 10% neutral-buffered formalin for 24 h. Following fixation, samples were collected and refixed in the same solution for another 24 h before undergoing gradual dehydration through an ethanol series (70–100%). Subsequently, the samples were cleared with xylene, embedded in paraffin, and sectioned into 4 μm-thick slices. The sections were then stained with hematoxylin-eosin (H&E) (BBC Biochemical, Washington, DC, USA) following standard protocols. 

The scoring of RSIV infection was divided into four stages: (1) less, (2) mild, (3) moderate, and (4) severe. Each stage was scored in blind experiments conducted by a fish pathologist. Renal and splenic lesions were assessed in two categories: enlarged cells and necrotic lesions. 

Enlarged cells in the spleen were scored from 1 to 4, depending on their frequency of occurrence in the parenchymal tissue. Enlarged cells in the kidney were evaluated based on the following criteria: score 1 (less) if present locally in renal tubules and parenchymal tissues; score 2 (mild) when a small number of cells were observed in the renal tubules, parenchymal tissues, and glomeruli. Necrotic lesions in the spleen were assessed based on these criteria: score 1 (less) for focal lesions involving a small number of cells; score 2 (mild) when numerous cells undergoing necrosis were identified; score 3 (moderate) when necrosis had progressed, cell exudate was observed, and inflammatory cell infiltration had occurred; and score 4 (severe) when necrosis had advanced, resulting in parenchymal tissue atrophy. Necrotic lesions in kidneys were evaluated based on the following criteria: score 1 (less) if localized and present in less than 5% of the renal tubule; score 2 (mild) if present locally and sporadically observed in 5% or more of the renal tubule; score 3 (moderate) if present in parenchymal tissues and glomeruli, in addition to renal tubules; and score 4 (severe) when tubule necrosis occurred due to necrotic lesions and atrophy resulting from parenchymal tissue and glomerular necrosis. The scoring standards are described in Figure S1.

The grading of RSIV-infected samples was achieved by weighting scores according to lesion severity. In brief, enlarged cells representing general lesions of RSIV infection accounted for 70% of the grading, while RSIV-induced necrotic lesions, which had a lower correlation with viral load, contributed to 30% of the grading. Changes in other organs were recorded, but not graded, due to their insignificant correlation. The grading scale employed was delineated as follows: Grade 0 (G0) included values less than or equal to 0.2; Grade 1 (G1) included values greater than 0.2 and less than or equal to 0.8; Grade 2 (G2) included values greater than 0.8 and less than or equal to 1.5; Grade 3 (G3) included values greater than 1.5 and less than or equal to 2.0; and Grade 4 (G4) included values exceeding 2.0.

### 2.7. Statistical Analysis

Statistical tests were performed using GraphPad Prism 9.5. An ordinary one-way analysis of variance (ANOVA) with Dunnett’s correction was performed when comparing multiple groups. Significant differences were compared with controls when the virus was first detected within each group. The correlation between histopathological infection grade and viral load was analyzed using Pearson correlation coefficients. Statistical significance was denoted by the following convention: * *p* < 0.05; ** *p* < 0.01; *** *p* < 0.001; **** *p* < 0.0001.

## 3. Results

### 3.1. Evaluation of Virulence of RSIV in Flathead Grey Mullets

#### 3.1.1. Mortality after RSIV Immersion Infection

**Figure 1:**
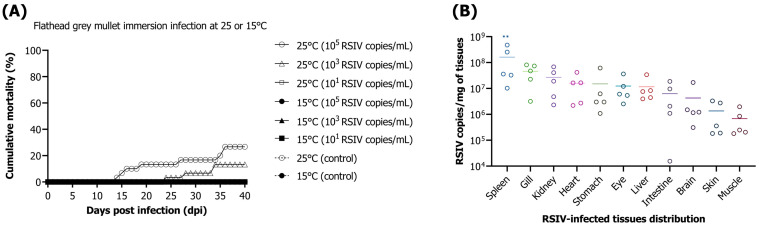
(**A**) Cumulative mortality of flathead grey mullet (*Mugil cephalus*) infection by immersion with red sea bream iridovirus (RSIV) at three concentrations (final concentrations 10^5^, 10^3^, and 10^1^ RSIV copies/mL) at 25 °C and 15 °C. The control group did not receive any treatment. (**B**) RSIV tissue distribution in flathead grey mullets that died due to RSIV. The bars represent the mean viral copy numbers (*n* = 5). Asterisks indicate significant differences (** *p* < 0.01) compared with the muscle.

The cumulative mortalities after immersion infection with 10^5^, 10^3^, and 10^1^ RSIV copies/mL in flathead grey mullets at 25 °C were 26.6%, 13.3%, and 0%, respectively (Figure 1A). At 15 °C, no mortality was observed in any immersion infection groups or in the negative control (Figure 1A). The distribution of RSIV in the tissues of the fish that died (*n* = 5, out of several fish that died) after immersion infection with 10^5^ RSIV copies/mL showed the highest viral load in the spleen (10^8.2^ RSIV copies/mg), which was significantly higher than the lowest viral load observed in the muscle (10^5.8^ RSIV copies/mg) (** *p* < 0.01) (Figure 1B).

#### 3.1.2. Viral Load Kinetics in Fish and Viral Shedding Ratio

**Figure 2:**
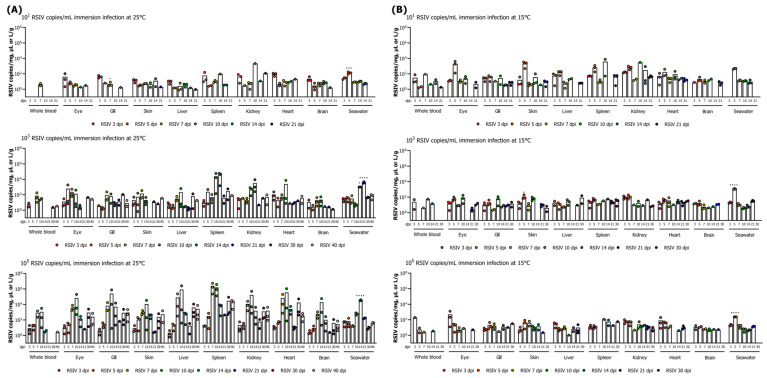
Viral load in various tissues (whole blood, eye, gill, skin, liver, spleen, kidney, heart, and brain) and RSIV shedding ratio in seawater after red sea bream iridovirus (RSIV) immersion infection in flathead grey mullets (*Mugil cephalus*) at (**A**) 25 °C and (**B**) 15 °C at three concentrations (final concentrations 10^5^, 10^3^, and 10^1^ RSIV copies/mL). The RSIV shedding ratio (viral genome copies L/g) was determined based on the total weight (g) of the fish remaining in the tank and the number of viral copies detected in the rearing seawater. Copy numbers of RSIV were analyzed in three fish and seawater per sampling interval. Significant differences were determined using one-way ANOVA with Dunnett’s multiple comparisons test (* *p* < 0.05; *** *p* < 0.001; **** *p* < 0.0001).

The immersion-infected groups with 10^5^ and 10^3^ RSIV copies/mL at 25 °C exhibited the highest viral loads (>10^4^ RSIV copies/mg) in the spleen at 7–10 dpi and 10–14 dpi, respectively (Figure 2A). After day 14, the viral loads decreased to the level of 10^2^–10^4^ RSIV copies/mg. In the immersion-infected group with 10^1^ RSIV copies/mL, a low viral load (<10^4^ RSIV copies/mg) was observed in all tissues until the end of the experiment, and RSIV was not detected in the fish after 21 dpi. Blood showed the lowest viral load at the three infectious concentrations. Higher concentrations of RSIV immersion infection correlated with higher virus detection rates and loads in fish. RSIV was detected in seawater from 3 dpi at all concentrations of immersion infection at 25 °C, and the viral load in the fish increased first, followed by an increase in the viral shedding ratio in seawater. In the immersion-infected group with 10^5^ and 10^3^ RSIV copies/mL, the highest RSIV shedding ratio in seawater was 10^4.5^ and 10^3.6^ RSIV copies L/g, respectively, at 14 dpi and 21 dpi, 2–3 days before fish mortality was observed.

In the RSIV immersion infection group at 15 °C, there was no difference in the viral load in the body according to the RSIV infection concentration, and no virus was detected after 30 dpi (Figure 2B). In addition, low infection loads were maintained in all tissues (<10^4^ RSIV copies/mg) until the end of the experiment. RSIV was detected in seawater from 3 dpi in the groups infected by immersion with 10^5^ and 10^3^ RSIV copies/mL at 15 °C, and RSIV was detected in seawater from 5 dpi in the immersion-infected group with 10^1^ RSIV copies/mL. The highest viral shedding ratios were observed in seawater at 5 dpi at all immersion concentrations (10^2.4^, 10^3.1^, and 10^2.7^ RSIV copies L/g net high immersion infection). Viral shedding ratios in seawater were observed until 21–30 dpi, similar to the time point at which no virus was detected in the fish.

### 3.2. Histopathological Analysis

#### 3.2.1. Histopathological Grade of RSIV Infection in Flathead Grey Mullets

**Figure 3:**
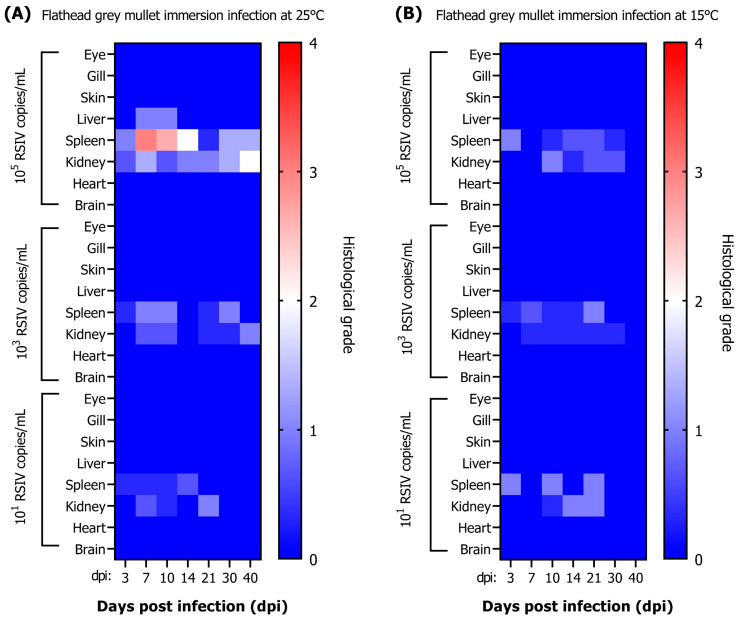
Histopathological grade results after immersion infection of flathead grey mullet (*Mugil cephalus*) with red sea bream iridovirus at three concentrations (final concentrations 10^5^, 10^3^, and 10^1^ RSIV copies/mL) at (**A**) 25 °C and (**B**) 15 °C. Each box represents the average of three fish.

After immersion infection of RSIV in flathead grey mullets at 25 °C, the viral load and histopathological infection grade were compared. Among the eight tissues of flathead grey mullet that were investigated, a specific infection grade change was identified in the spleen, and the specific lesion of RSIVD was identified only in the spleen and kidney (Figure 3A). In the other two fish, lesions were confirmed in the liver. In the immersion-infected group with 10^5^ RSIV copies/mL, G2–G3 lesions were identified on days 7–10, with a high viral load in the spleen (10^5.9^–10^6.1^ RSIV copies/mg) (Figure 2A and Figure 3A). Subsequently, the histopathological grade decreased as the viral load decreased. Lesions graded lower than G2 were observed in the spleen and kidney in the immersion-infected groups with 10^1^ and 10^3^ RSIV copies/mL. In the group infected with 10^1^ RSIV copies/mg, an infection grade of G0 was observed, as no virus was detected after 30 dpi. In the RSIV immersion-infection group at 15 °C, lesions lower than G2 were observed at all infection concentrations, and RSIVD-specific histopathological lesions were observed only in the spleen and kidney (Figure 3B).

#### 3.2.2. Correlation between Viral Load and Histopathological Infection Grade

**Figure 4:**
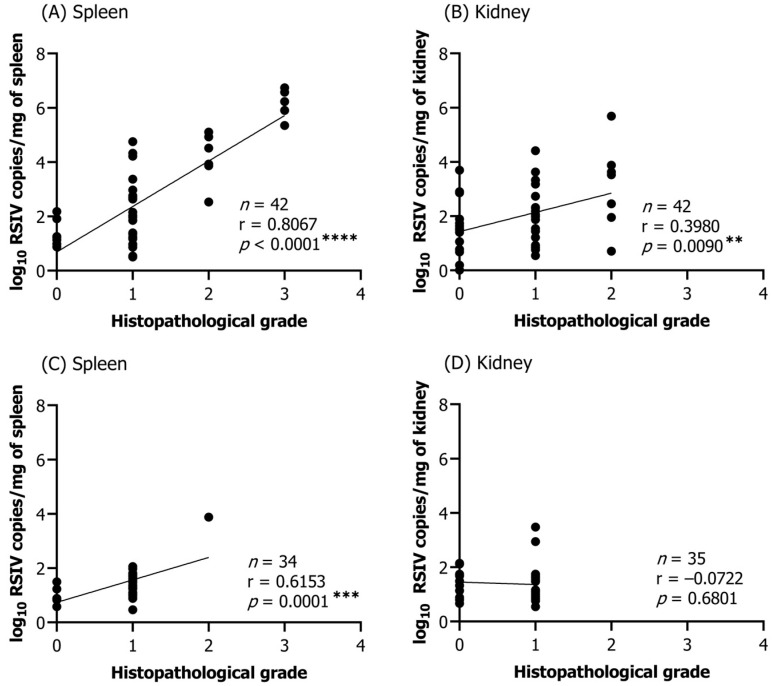
Correlations between the RSIV viral load and histopathological infection grade in the spleen and kidney of flathead grey mullets (*Mugil cephalus*) infected by RSIV immersion at (**A**,**B**) 25 °C and (**C**,**D**) 15 °C. Statistical significance was determined using Pearson correlation coefficients (** *p* < 0.01; *** *p* < 0.001; **** *p* < 0.0001).

Correlations between the viral load and infection grade were analyzed only among RSIV immersion-infected flathead grey mullets at 25 °C and 15 °C. RSIVD-specific lesions were observed only in the spleen, kidney, and liver; however, the liver was excluded from further analysis due to the low detection rate of histopathological lesions and the insignificant correlation with the viral load of RSIV. An analysis of correlations between viral load and infection-grade was performed in selected samples in which RSIV was detected by qPCR in the spleen and kidneys of the immersion-infected flathead grey mullets. At 25 °C, a significant correlation was observed between viral load and infection grade in both the spleen and kidney (spleen: r = 0.8067, **** *p* < 0.0001; kidney: r = 0.3980, ** *p* = 0.0090) (Figure 4A,B). At 15 °C, a significant correlation was only detected in the spleen (spleen: r = 0.6153, *** *p* = 0.0001; kidney: r = −0.0722, *p* = 0.6801) (Figure 4C,D). Among them, a higher histopathological infection grade (G3) was observed in the spleen rather than in the kidney, and a significantly higher positive correlation between viral load and infection grade was observed (Figure 4).

### 3.3. Cohabitation Challenge

#### 3.3.1. Cumulative Mortality after Cohabitation Challenge

**Figure 5:**
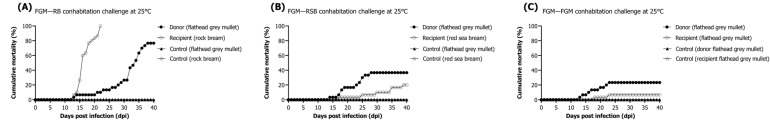
Cumulative mortality after red sea bream iridovirus (RSIV) cohabitation challenge between rock bream (*Oplegnathus fasciatus*), red sea bream (*Pagrus major*), and flathead grey mullet (*Mugil cephalus*) at 25 °C. (**A**) Cumulative mortality after cohabitation of naïve rock bream (recipient) with flathead grey mullet (donor) intraperitoneally (IP) injected with RSIV (10^6^ RSIV copies/fish) at 25 °C. (**B**) Naïve red sea bream. (**C**) Naïve flathead grey mullet: cumulative mortality after cohabitation with IP-injected flathead grey mullet (donor). The control group (donor) was IP injected with 100 uL of L-15 medium (virus-free), and the recipient was untreated. Mortality was not observed at 15 °C.

An interspecies cohabitation challenge was conducted to evaluate the risk of horizontal transmission in RSIV-infected flathead grey mullets. Cumulative mortality was assessed after cohabitation challenges between flathead grey mullets (donors) and rock bream, red sea bream, and flathead grey mullets (recipients). At 25 °C, flathead grey mullets (donors) showed a 76.6% cumulative mortality up to 40 dpi, while rock bream (recipients) had 100% cumulative mortality at 22 dpi (Figure 5A). The cumulative mortalities after the cohabitation challenge between flathead grey mullets (donors) and red sea bream (recipients) were 36.6% and 20% (Figure 5B), respectively, lower than those observed for flathead grey mullets (donors) and rock bream (recipients). Flathead grey mullets (donors) and (recipients) showed the lowest cumulative mortalities at 23.3% and 6.7% (Figure 5C), respectively. No deaths were observed in any groups challenged with cohabitation at 15 °C, or in the negative control groups at 25 °C and 15 °C (Figure 5).

#### 3.3.2. Viral Dynamics in Fish and Rearing Seawater within Cohabitation Challenge

**Figure 6:**
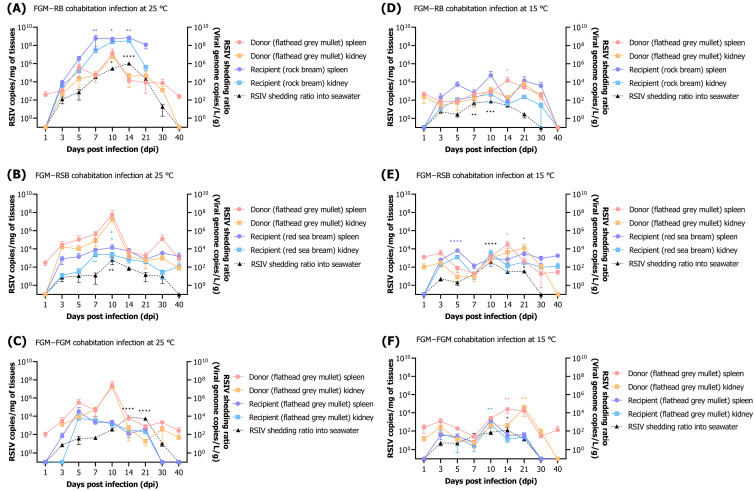
(**A**–**F**) Viral kinetics after cohabitation challenges involving naïve rock bream (*Oplegnathus fasciatus*), red sea bream (*Pagrus major*), and flathead grey mullet (*Mugil cephalus*) post red sea bream iridovirus (RSIV) intraperitoneal injection (10^6^ RSIV copies/fish) in flathead grey mullet at 25 °C and 15 °C. Viral load in fish was measured in the spleen and kidney, and virus shed from fish into rearing seawater was expressed as RSIV shedding ratio. The RSIV shedding ratio (viral genome copies L/g) was determined based on the total weight (g) of the fish remaining in the tank and the number of viral copies detected in the rearing seawater. Copy numbers of RSIV were analyzed in three fish and seawater per sampling interval. Significant differences were determined using one-way ANOVA with Dunnett’s multiple comparisons test (* *p* < 0.05; ** *p* < 0.01; *** *p* < 0.001; **** *p* < 0.0001).

RSIV-infected flathead grey mullets (donors) at 25 °C showed the highest viral load (10^7.2^ RSIV copies/mg) in the spleen at 10 dpi, and the load continued to decrease in the spleen and kidney after 14 dpi (Figure 6A). Rock bream (recipients) showed a higher viral load (10^8.8^ RSIV copies/mg) in the spleen at 7 dpi, which was significantly higher than that of rock bream (recipient; at 3 dpi; ** *p* < 0.01). In seawater at 25 °C, the virus showed the highest RSIV shedding ratio (10^6.0^ RSIV copies L/g) at 14 dpi, where mortality of rock bream (recipient) was observed. Subsequently, the virus in seawater decreased to 10^1.3^ RSIV copies L/g at 30 dpi after 100% mortality of rock bream (recipient) (Figure 6A). In the cohabitation challenge group of flathead grey mullet and rock bream at 15 °C, the highest viral load (10^4.1^ RSIV copies/mg) was observed in the spleen at 14 dpi after the RSIV inoculation of the flathead grey mullet (donor) (Figure 6D). Viral load was observed at a lower level (<10^5^ RSIV copies/mg) than in the 25 °C group in the rock bream (recipient). The virus in seawater was 10^1.8^ RSIV copies L/g at 10 dpi, a low viral shedding ratio compared with the 25 °C group.

In the cohabitation challenge group between flathead grey mullet and red sea bream at 25 °C, the viral load in the spleen peaked at 10^7.8^ RSIV copies/mg at 10 dpi in flathead grey mullet (donor) (Figure 6B); after 14 dpi, it continuously decreased. The virus was detected from 3 dpi in red sea bream (recipient) and maintained at a low level (10^4^ RSIV copies/mg) until the end of the experiment (40 dpi). The RSIV shedding ratio in seawater peaked at 10 dpi (10^2.8^ RSIV copies L/g) and was not detected at 40 dpi (Figure 6B). The highest load of virus in the spleen, 10^4.5^ RSIV copies/mg, was detected at 14 dpi after RSIV inoculation of flathead grey mullet (donor) at 15 °C (Figure 6E). In red sea bream (recipient), the virus was detected at 3 dpi, and viral loads of less than 10^4^ RSIV copies/mg were observed at all sampling intervals. The virus in seawater was the highest, with 10^2.5^ RSIV copies L/g at 10 dpi, and was not detected from 30 dpi (Figure 6E).

In a cohabitation challenge experiment between flathead grey mullet and flathead grey mullet at 25 °C, the viral load in the spleen at 10 days after RSIV infection in flathead grey mullet (donor) was the highest at 10^7.4^ RSIV copies/mg (Figure 6C). After 14 dpi, it continued to decrease, similar to the results of cohabitation challenges with other fish species (rock bream and red sea bream). In the flathead grey mullet (recipient), the highest viral load of 10^4.5^ RSIV copies/mg was observed in the spleen at 5 days after exposure to the virus, which subsequently decreased continuously, and the virus was not detected at 30 dpi. The highest RSIV shedding ratio (10^3.9^ RSIV copies L/g) in seawater at 25 °C was found at 14 dpi, which coincided with the time when flathead grey mullet (donor) mortality was observed (Figure 6C). After the RSIV infection of the flathead grey mullet (donor) at 15 °C, the highest viral load of 10^4.4^ RSIV copies/mg was detected in the spleen at 14 dpi, and no virus was detected in the kidneys from 40 dpi (Figure 6F). In the case of the flathead grey mullet (recipient), RSIV was detected due to horizontal transmission, but a low viral load (<10^4^ RSIV copies/mg) was observed, and the virus was not detected at 30 dpi (Figure 6F).

#### 3.3.3. Histopathological Infection Grade

**Figure 7:**
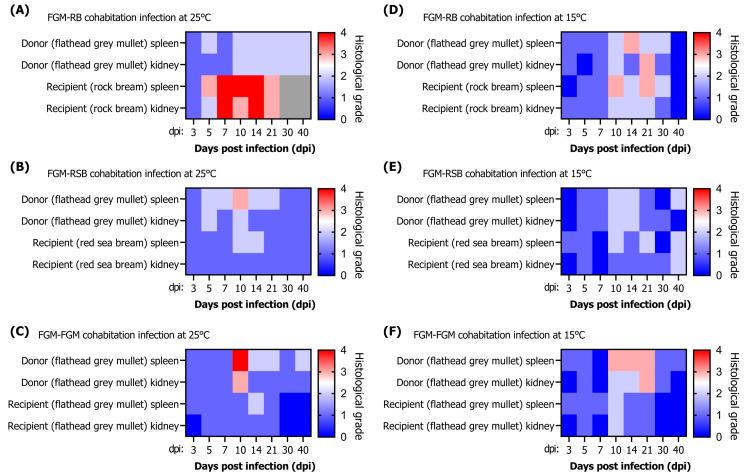
(**A**–**F**) Histopathological grade results after red sea bream iridovirus (RSIV) cohabitation challenge involving rock bream (*Oplegnathus fasciatus*), red sea bream (*Pagrus major*), and flathead grey mullet (*Mugil cephalus*) at 25 °C and 15 °C. The experiment was repeated thrice, and the analysis was not performed because all fish died due to RSIV infection, as seen in the gray box of (**A**).

In the cohabitation challenge between flathead grey mullet and rock bream at 25 °C, a G2 lesion was observed at 10 dpi with a high viral load in the spleen (10^7.2^ RSIV copies/mg) in the flathead grey mullet (donor) (Figure 7A). In the rock bream (recipient), G3–G4 lesions were observed in the spleen and kidney at 7–14 dpi, and the viral load was approximately 10^8^ RSIV copies/mg. At 15 °C, a G3 lesion was observed in the spleen at 14 dpi in the flathead grey mullet (donor), and a G3 lesion in the spleen was observed at 10 dpi and at 21 dpi in the rock bream (recipient) (Figure 7D).

As a result of the cohabitation challenge between a flathead grey mullet and red sea bream at 25 °C, G3 lesions were observed at 10 dpi, when a high viral load in the spleen was observed in the flathead grey mullet (donor); further, G1–G2 lesions were observed as the viral load decreased (Figure 7B). In the red sea bream (recipient), G2 and G1 lesions were observed at 10–14 dpi and 21–40 dpi, respectively. At 15 °C, G2 lesions were observed in the spleen at 10–14 dpi in the flathead grey mullet (donor), and G2 lesions were observed in the spleen at 10 dpi and 21 dpi in the red sea bream (recipient) (Figure 7E).

In the cohabitation challenge between flathead grey mullet and flathead grey mullet at 25 °C, a G4 lesion was observed at 10 dpi, which showed a high viral load (10^7.4^ RSIV copies/mg) in the spleen of the flathead grey mullet (donor), following which the viral load and infection grade decreased (Figure 7C). The flathead grey mullet (recipient) showed G2 lesions in the spleen at 14 dpi, followed by low-grade lesions. At 15 °C, G3 lesions were observed in the spleen at 10–21 dpi in the flathead grey mullet (donor), and G2 lesions were observed in the spleen and kidney at 10 dpi in the flathead grey mullet (recipient) (Figure 7F).

## 4. Discussion

The goal of this study was to demonstrate pathogenicity to mullet using immersion infection, similar to the mechanism of natural RSIV infection in fish farms. To understand the mechanisms of viral load, viral shedding, and histopathological lesions in RSIV-infected flathead grey mullets, challenge experiments were performed at various infection concentrations and water temperatures. In addition, a cohabitation challenge model was used to assess the risk of horizontal transmission of RSIV. To the best of our knowledge, this is the first report of RSIV kinetic analysis and cohabitant infection in flathead grey mullets.

In the current study, the mortality of flathead grey mullets challenged by immersion infection was observed at concentrations of 10^5^ and 10^3^ RSIV copies/mL. Previous research has shown that Japanese amberjack (*Seriola quinqueradiata*) subjected to immersion challenge with RSIV (10^2.7^ TCID_50_/mL) at 25 °C experienced a 40% mortality rate [27]. In Pacific bluefin tuna (*Thunnus orientalis*), a 44.9% mortality rate was reported when immersion-infected at 25 °C with a concentration of 6.0 × 10^3^ RSIV copies/mL [28]. In rock bream, 100% mortality occurred when challenged by immersion with 10^1^, 10^3^, 10^5^, and 10^7^ RSIV copies/mL [16]. Therefore, mortality in fish challenged by immersion with 10^5^ RSIV copies/mL in this study was the lowest at 26.6% compared with those reported in previous research [16,27,28]. Susceptibility to RSIV may vary among species, but the impact of fish species with relatively low susceptibility to disease on other species can be significant in the aquaculture industry. RSIVD is known to occur mainly at water temperatures above 20 °C, with an optimal temperature of approximately 25 °C [1,15,29]. In several studies, the highest viral shedding has been reported to occur just before or during periods of active mortality following exposure to the virus [15,16,17,27,30]. In this study, a low mortality rate was observed following the RSIV immersion challenge; however, the peak of virus shedding was observed either during the period of mortality or 3–4 days prior to the occurrence of mortality. These observations suggest that the virus shed from flathead grey mullets may potentially cause horizontal transmission to other fish species. Interestingly, viral shedding was detected from 3 to 5 days in flathead grey mullets that did not exhibit mortality. A similar trend in dynamics was observed in rock bream challenged with RSIV at 15 °C without mortality [15]. Additionally, in seven-band grouper (*Hyporthodus septemfasciatus*) infected with nervous necrosis virus (NNV), viral shedding was detected within 24 h [31], while in Japanese amberjack infected with RSIV, the virus was found at 3 dpi (10^2.5^ RSIV genome copies/L) [27]. In Atlantic salmon (*Salmo salar* L.), excretion of salmonid alphavirus (SAV) through mucus and feces is known to begin immediately after infection and may persist for 3–4 weeks thereafter [32]. Although this study did not analyze viral loads in feces and mucus, the virus was consistently detected during the initial stages of infection and at low water temperatures for 14–21 days. However, no differences were observed in the viral loads within the fish and the amounts of virus shed into the seawater, even at high RSIV infection concentrations in low water temperatures. While the precise conditions for virus release into seawater cannot be determined, the virus may be shed when an apparent infection is induced within the fish population. However, no RSIV was detected in seawater after 21–30 dpi in water temperatures at which no disease progression was observed. These results are consistent with those of previous studies that showed that viral shedding terminates as viral load declines and RSIV-infected rock bream recover at water temperatures as low as 15 °C [15,33]. In the present study, because the challenge experiments were performed in a closed aquaculture system rather than a flow-through aquaculture system, we may not have fully reproduced the disease progression in the aquaculture field. Despite replacing 50% of the tank seawater daily, it cannot be ruled out that the accumulation of virus shed from the fish may have occurred, potentially leading to an overestimation of viral shedding. However, in a previous study, even when a large amount of rearing seawater in the tank was flushed with fresh seawater after virus infection in fish, the time points for the viral load detection in the fish and the peak of viral load in the seawater were consistent [27]. Our results are the first to demonstrate the establishment of RSIV infection in flathead grey mullets in an immersion infection model that mimics naturally occurring infections. However, conditions in a fish farm environment may differ from those of temporary immersion infection in a laboratory environment, as RSIV present in rearing seawater has the possibility of continuously infecting fish.

The spleen and kidneys are known to be the main target organs for RSIV, but RSIV can also be detected in the gills, heart, and intestines [1,25]. In our study, the highest viral load was observed in the spleen in dead flathead grey mullets by RSIV, followed by the gills, kidneys, and heart. In flathead grey mullets challenged by immersion infection at a water temperature of 25 °C (in which RSIV-susceptibility was observed), the RSIV load was the highest in the spleen among all organs tested during most stages from the initial infection to the recovery period. However, in the group without mortality or disease progression (10^1^ RSIV copies/mL at 25 °C and all challenge groups at 15 °C), a low viral load was observed in all organs examined regardless of the RSIV target organ. Based on these results, our study strongly suggests that the spleen is the most suitable target organ for the detection of RSIV in flathead grey mullets under disease-progression conditions.

Histopathological lesions in fish can indicate the severity and progression of a disease. Several studies have investigated the correlation between viral loads and histopathological lesions. However, a positive PCR reaction does not necessarily imply ongoing disease progression in fish. In a previous study, RSIV was detected in cultured rockfish (*Sebastes schlegelii*) by nested PCR, but no histopathological lesions were observed in the tissues [34]. In another study, rock bream that survived RSIV infection were reported to still be detectable by PCR even after 100 days [20]. In our study, histopathological lesions were primarily observed in the spleen and kidney, but some fish tissues with low viral loads did not exhibit enlarged cells or necrotic lesions. This may be due to insufficient initial replication time, inappropriate replication temperature, or inadequate viral infection capacity, resulting in no observable lesions. The formation of abnormally enlarged cells due to RSIV infection is frequently observed in the spleen of infected fish [35]. In our study, a high correlation between viral load and lesions was confirmed in the spleen of infected flathead grey mullets. The parenchymal tissue of the spleen in fish with high viral loads was replaced by enlarged cells. Necrotic lesions tended to be observed earlier than enlarged cells as a more sensitive primary response at low viral loads. In contrast to the observations for the spleen, a low correlation between lesions in the kidney and viral load was demonstrated. The impact of abnormally enlarged cells on kidney function might be minimal, as these cells only appear on interstitial hematopoietic tissue and did not occupy the renal parenchymal tissue, which is consistent with previous studies [2]. As there are few reports describing the histopathology of diseased flathead grey mullets, our findings could contribute to future research in fish pathology. Additionally, it is crucial to consider both viral loads and histopathological lesions in order to accurately assess disease status in infected fish.

In previous studies, research on RSIV cohabitation challenges between rock bream and rockfish has been reported [13]; however, there is no literature regarding flathead grey mullets. A cohabitation challenge model was used to assess whether RSIV-infected flathead grey mullets develop horizontal transmission to recipients. Mortality patterns and viral shedding kinetics differed according to the RSIV susceptibility of recipient species cohabiting with flathead grey mullets. The timing of the first mortality of mullet in each challenged group at 25 °C was similar, at 13–14 dpi. The highest cumulative mortality rate was observed in groups cohabitating with rock bream (recipients). These results are thought to be the cause of the high mortality and viral shedding rate, as the rock bream infected with the virus shed by the flathead grey mullet had the highest RSIV susceptibility [1]. Nevertheless, in our findings, the viral load of flathead grey mullet (donor) was at a similar level in each group within 21–40 days post-infection at 25 °C, but the mortality rates differed. These discrepancies may be due to the tanks used for mortality measurement being different from those sampled for viral load measurement or because the collected live fish were gradually recovering from the disease. The viral load and mortality trends may have differed as a result. After the cohabitation challenge, all deceased fish exhibited an RSIV load higher than approximately 10^7^ RSIV copies/mg (data not shown). Lower mortality and viral shedding ratios were observed in the red sea bream and flathead grey mullet groups (recipients). This is consistent with previous research findings suggesting that red sea bream has a lower susceptibility to RSIV compared with rock bream [1]. Additionally, this aligns with our experience that, in Korean fish farms, mortality due to RSIVD is less frequently observed in these two species than in rock bream. Histopathological lesions were also observed in the cohabitation challenge experiment. Although the histopathological grading of donors and recipients did not show a perfect correlation with viral load, the infection grade followed as the viral load increased. In particular, flathead grey mullets infected at lower water temperatures shed the virus into the seawater, and horizontal transmission was confirmed as the virus was detected in healthy fish. Additionally, the progression of the disease was demonstrated through the observation of lesions within recipient fish tissues. These results suggest that the virus shed from RSIV-infected flathead grey mullets can move through seawater and damage nearby farmed fish such as rock bream, red sea bream, and flathead grey mullets, which are all farmed in open-net pens in Korea.

## 5. Conclusions

In conclusion, the results of this study indicate that flathead grey mullets infected with RSIV can release the virus into the seawater, and there is a high correlation between viral load and histopathological lesions. The current cohabitation study demonstrates that viral transmission can primarily occur during the initial exposure and, notably, highlights the potential for RSIV to spread to other fish species even at lower water temperatures. However, further research on the infection dynamics of different RSIV genotypes in other host species is crucial for understanding the virus’s infectivity and adaptability.

## Data Availability

The data presented in this study are available upon request from the corresponding author.

## Supplementary Materials

The following supporting information can be downloaded at: https://www.mdpi.com/article/10.3390/ani13081341/s1, Figure S1: Scoring of lesions: Histopathological scoring of the lesions of red sea bream iridovirus-induced necrotizing lesions and enlarged cell lesions in the flathead grey mullet (*Mugil cephalus*) spleen and kidney tissues (hematoxylin and eosin stain, bar = 50 μm).
