# Supplementary material for: Red Sea Bream Iridovirus Kinetics, Tissue Tropism, and Interspecies Horizontal Transmission in Flathead Grey Mullets (Mugil cephalus)
Source: Animals (Basel). 2023 Apr 13;13(8):1341. doi: 10.3390/ani13081341 (PMC10135084; doi:10.3390/ani13081341)
Supplement: Supplementary file 1 [file animals-13-01341-s001.zip › animals-2335675-supplementary.pdf]

animals

MDPI

Supplementary Material

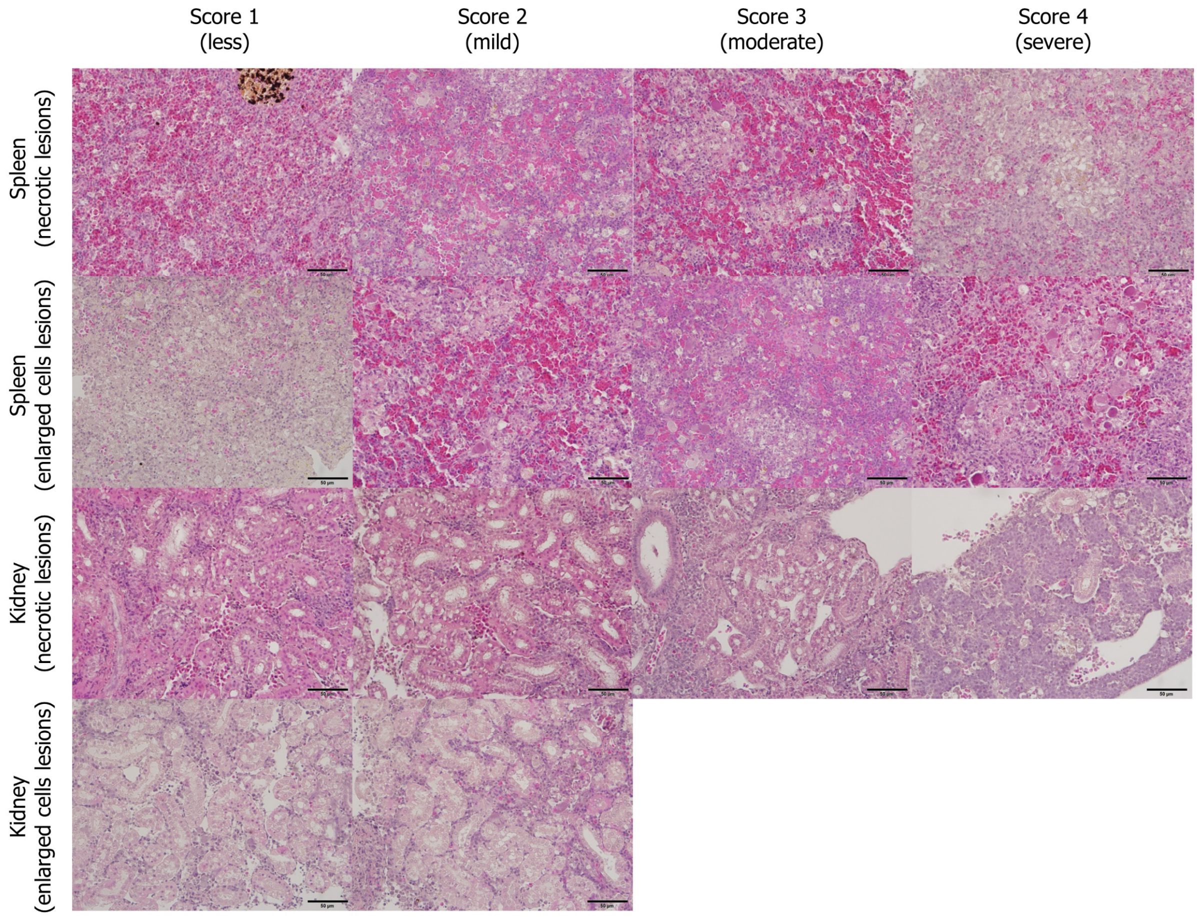

Figure S1. Scoring of lesions: Histopathological scoring of the lesions of red sea bream iri-dovirus-induced necrotizing lesions and enlarged cells lesions in the flathead grey mullet (*Mugil cephalus*) spleen and kidney tissues (haematoxylin and eosin stain, bar = 50 µm).
